# Association of intimate partner violence during pregnancy, prenatal depression, and adverse birth outcomes in Wuhan, China

**DOI:** 10.1186/s12884-018-2113-6

**Published:** 2018-12-03

**Authors:** Honghui Yu, Xueyan Jiang, Wei Bao, Guifeng Xu, Rong Yang, Min Shen

**Affiliations:** 1 Department of Anesthesiology, Tongji Hospital affiliated Tongji Medical College, Huazhong University of Science and Technology, Wuhan, 430030 China; 2 Department of Maternal and Child Health, School of Public Health, Tongji Medical College, Huazhong University of Science and Technology, Wuhan, 430030 China; 3 Department of Epidemiology, College of Public Health, University of Iowa, Iowa City, IA 52242 USA; 4 Center for Disabilities and Development, University of Iowa Stead Family Children’s Hospital, Iowa City, IA 52242 USA; 5 Wuhan Children’s Hospital (Wuhan Maternal and Child Healthcare Hospital), Tongji Medical College, Huazhong University of Science & Technology, Wuhan, 430015 China

**Keywords:** Intimate partner violence, Prenatal depression, Adverse birth outcome, Pregnancy

## Abstract

**Background:**

Intimate partner violence (IPV) among pregnant women constitutes a global public health problem and a potential risk factor for adverse maternal and fetal outcomes. The present study aimed to examine the associations among IPV during pregnancy, prenatal depression, and adverse birth outcomes in Wuhan, China.

**Methods:**

A cross-sectional study was performed from April 2013 to March 2014 in Wuhan, China. Sociodemographic characteristics, IPV during pregnancy, and depressive symptoms during pregnancy were assessed in the third trimester of pregnancy. Birth outcomes were collected after delivery using medical records. Chi-square tests and logistic regression analysis were used to examine the association between IPV and prenatal depression, as well as the association between IPV combined with prenatal depression and adverse birth outcomes.

**Results:**

After adjustment for covariates, there was a statistically significant association between IPV during pregnancy and prenatal depression (adjusted odds ratio [aOR] = 2.50, 95% confidence interval [CI]: 1.60–3.90). IPV during pregnancy (aOR = 1.67, 95% CI: 1.08–2.56) and prenatal depression (aOR = 1.72, 95% CI: 1.11–2.68) were significantly associated with adverse birth outcomes. Women experiencing psychological abuse had a significantly higher odds of prenatal depression (aOR = 2.04, 95% CI: 1.19–3.49) and of adverse birth outcomes (aOR = 2.13, 95% CI: 1.08–2.58), compared with women who did not experience IPV and prenatal depression.

**Conclusions:**

IPV during pregnancy and prenatal depression were significantly associated with adverse birth outcomes, after adjustment for socio-demographic and behavior factors. The findings suggest that early recognition of IPV and prenatal depression during antenatal care may protect pregnant women and improve birth outcomes.

## Background

Intimate partner violence (IPV) among pregnant women constitutes a global public health problem [1, 2] and is a potential risk factor for adverse maternal and fetal outcomes [3, 4]. IPV includes physical abuse, psychological abuse, sexual violence, and economic abuse in the home setting [5, 6]. A study conducted by World Health Organization [7] based on household data from the Demographic and Health Surveys (DHS) and the International Violence Against Women Survey (IVAWS) across 19 countries showed that the prevalence of physical IPV during pregnancy was 3.8~ 13.5% in Africa, 4.1~ 11.1% in Americas, 1.8~ 6.6% in Europe and 2.0–5.0% in Asia. Previous studies have suggested that IPV disproportionately affects low-income women, especially for pregnant women [8]. A previous survey showed that the prevalence of domestic violence among pregnant women was 15.9% in Japan [9], 6.5% in the United States [10], 7.7% in Spain [11], and 34.8% in northern European countries [12]; however, the prevalence rates were much higher in less developed countries such as Iran (72.8%) [13] and Nigeria (44.6%) [14]. In China, the prevalence rates of IPV among pregnant women have been reported as 18.8% in Hong Kong [15] and 11.3% in Changsha [16].

IPV during pregnancy does not only affect women’s health; it also has adverse health effects for newborns and affects their development in childhood [17–19]. Previous studies have demonstrated a relationship between abuse during pregnancy and both low birth weight (LBW) and preterm birth (PTB) [13, 18–20]. Many studies have shown that pregnancy constitutes a particularly critical period because of women’s increased vulnerability and body changes, increased economic pressure, and less frequent sexual relations [18, 21, 22]. Pregnant women experiencing IPV also experience depression and anxiety. Studies have shown that women experiencing abuse during pregnancy are 2.5 times more likely to report depressive symptomatology, compared with their non-abused counterparts [23, 24]. These symptoms may be the direct consequence trauma or the indirect consequence of domestic violence [25–27].

In China, little is known about the experience of IPV among pregnant women or about its impacts on mental health and on children. Therefore, this study aimed to investigate the association between IPV during pregnancy and prenatal depression and the associations of these variables with adverse birth outcomes, adjusting for covariates.

## Methods

This study was carried out in Qiaokou District Maternal and Child Health Hospital, Dongxihu District People’s Hospital, and Tongji Hospital from April 1, 2013, to March 31, 2014, in Wuhan, China. The participants were pregnant women attending prenatal examinations or delivery at the selected hospitals during the study period. All participants were assessed in the third trimester of pregnancy or prior to delivery. This study was approved by the Ethics Board of Tongji Medical College, Huazhong University of Science and Technology. Written informed consent was obtained from the participants.

The Abuse Assessment Screen (AAS) was used to assess IPV during pregnancy [28]. Women reporting yes on any of the IPV items for the period of the current pregnancy were defined having been exposed to IPV. The Chinese version of AAS was first used in 1999 by Leung to screen for IPV among Chinese women [28]. The Chinese AAS has demonstrated a satisfactory level of measurement accuracy, with high specificity (≥ 89%) and positive predictive values (≥ 80%) and satisfactory-to-high negative predictive values (66–93%) [29]. IPV was defined as physical abuse, psychological abuse, sexual abuse, or economic abuse. Prenatal depression was measured using the Center for Epidemiologic Studies-Depression Scale (CES-D) [30]. The CES-D is a 20-item self-report scale designed to measure current levels of depressive symptoms and has been widely used in China. The Cronbach’s α of the CES-D overall has been shown to be 0.90, and it is 0.68–0.86 for each of the scale’s factors [31]. CES-D scores range from 0 to 60, with higher scores signifying more severe symptoms of depression. The participants were considered likely to be depressed if their scores were ≥ 20.

Data were collected on the demographic characteristics of pregnant women and their partners, husbands’ smoking and alcohol use behaviors, family status, and pregnancy-related complications. Information on birth outcomes and pregnancy-related complications was collected from medical records after delivery. Adverse birth outcomes included PTB, LBW, birth defects, asphyxia, and stillbirth. PTB was defined as birth prior to 37 weeks’ gestation, following the definition of the World Health Organization [32]. Using the typical definition, stillbirth was defined as fetal death at or after 20 to 28 weeks of pregnancy [32]. LBW was defined using the World Health Organization’s definition of an infant having a birth weight of 2499 g or less, regardless of gestational age. “Birth defect” is a phrase commonly used to describe congenital malformations (i.e., a congenital or physical anomaly that is recognizable at birth) [33]. Perinatal asphyxia is the medical condition resulting from a newborn infant being deprived of oxygen (hypoxia) for long enough to cause apparent harm. Miscarriage was defined as an early pregnancy loss, and live birth was defined as the baby being born alive, even if he/she died shortly afterward.

All statistical analyses were performed using SAS software, Version 9.4 (SAS Institute Inc., Cary, North Carolina). Descriptive statistics were used to calculate the prevalence of overall IPV and subtypes of IPV, including physical, psychological, and sexual abuse—categorical variables described by frequency distributions. Chi-square tests were used for bivariate analyses to assess differences in the prevalence rates of IPV. Continuity-adjusted chi-square analysis was applied when 25% of the cells had expected counts of less than five. Multivariable logistic regression was used to estimate the adjusted odds ratios (ORs) and 95% confidence intervals (CIs) for differences between the abused group and the non-abused group. All statistical tests were two-sided, and significance was determined using an alpha level of 0.05.

## Results

A total of 900 pregnant women participated in the survey. Of these women, 797 returned questionnaires that were eligible for data analysis. The average maternal age was 27.4 ± 4.2 years. More than one-third of the participants had received a bachelor’s degree or higher, and more than half had monthly household incomes of less than 6000 yuan (Table 1).

**Table 1:** Characteristics of pregnant women participated in the survey in Wuhan, China

Variables	IPV (%)	No IPV (%)	χ^2^ value	*P* value
Age (years)				
20–24	43 (20.00)	172 (80.00)	0.6547	0.8838
25–29	62 (17.42)	294 (82.58)		
30–34	33 (18.44)	146 (81.56)		
35+	8 (17.02)	39 (82.98)		
Maternal education level^**^				
Junior middle school or below	30 (24.59)	92 (75.41)	17.2640	0.0006
High school	52 (25.49)	152 (74.51)		
Vocational degree	25 (13.66)	158 (86.34)		
Bachelor degree or above	39 (13.54)	249 (86.46)		
Maternal occupation				
Employed	70 (14.68)	407 (85.32)	10.5410	0.0012
Unemployed	76 (23.75)	244 (76.25)		
Education level of husband				
Junior middle school or below	31 (27.68)	81 (72.32)	9.7016	0.0213
High school	39 (19.50)	161 (80.5)		
Vocational degree	30 (17.65)	140 (82.35)		
Bachelor degree or above	46 (14.60)	269 (85.4)		
Occupation of husbands				
Employed	126 (18.89)	541 (81.11)		
Unemployed	20 (15.38)	110 (84.62)		
Household income monthly (Yuan)				
> 3000	43 (25.6)	125 (74.4)	0.8937	0.3445
3000-	61 (19.55)	251 (80.45)		
6000-	28 (14.66)	163 (85.34)		
≥ 10,000	14 (11.11)	112 (88.89)		
Abortion history^**^				
Yes	66 (23.9)	210 (76.1)	8.8310	0.0030
No	80 (15.4)	441 (84.6)		
Parity^**^				
1	67 (14.5)	396 (85.5)	11.9798	0.0025
2	44 (21.9)	157 (78.1)		
≥ 3	35 (26.3)	98 (73.7)		
Husband’s smoking behavior				
Yes	80 (19.4)	332 (80.6)	0.6882	0.4068
No	66 (17.1)	319 (82.9)		
Husband’s drinking behavior				
Yes	113 (17.4)	537 (82.6)	2.0550	0.1517
No	33 (22.4)	114 (77.6)		
Pregnancy related complications				
Yes	31 (19.38)	129 (80.62)	0.1493	0.6992
No	115 (18.05)	522 (81.95)		
Adverse birth outcomes				
Yes	39 (26.53)	108 (73.47)	8.1744	0.0042
No	107 (16.44)	544 (83.56)		
Prenatal depression				
Yes	43 (32.58)	89 (67.42)	21.4908	<.0001
No	103 (15.49)	562 (84.51)		
Total	146 (18.32)	651 (81.68)		

A total of 146 (18.32%) of the pregnant women reported that they had experienced IPV by their husbands during the pregnancy. The prevalence rates of psychological, physical, economic, and sexual abuse were 14.3%, 2.1%, 2.0%, and 0.3%, respectively. The overall prevalence rate of adverse birth outcomes was 18.44% (147/797). The prevalence rates of neonatal asphyxia, PTB, and LBW were 12.3%, 8.9%, and 5.3%, respectively. The women who had adverse birth outcomes had a higher rate of IPV during pregnancy than did women without adverse birth outcomes (26.53% vs. 16.44%, *P* = 0.0044). The IPV group had a higher prevalence rate of depression than did the non-IPV group (32.58% vs. 15.49%, *P* < 0.001) (Table 1).

Table 2 showed the differences of prevalence rates among IPV group and no IPV group. The results suggested that women experienced IPV had higher rates of prenatal depression than those no IPV (*P* < 0.0001). For subtype of IPV, women experienced psychological, psychological and physical abuse had significant higher prevalence rates of prenatal depression (*P* < 0.01).

**Table 2:** The differences of prevalence rates of depression among the IPV or the type of IPV group compared to no IPV group

IPV or subtype of IPV	Prenatal depression (%)	No prenatal depression (%)	χ^2^ value	*P* value
Overall IPV			21.4908	<.0001
No	89 (13.67)	562 (86.33)		
Yes	43 (29.45)	103 (70.55)		
Psychological abuse			7.7637	0.0053
No	107 (15.22)	596 (84.78)		
Yes	25 (26.60)	69 (73.40)		
Psychological + physical abuse			17.1939^a^	<.0001
No	125 (15.88)	662 (84.12)		
Yes	7 (70.00)	3 (30.00)		
Psychological+ economic abuse			3.2836^a^	0.0700
No	128 (16.24)	660 (83.76)		
Yes	4 (44.44)	5 (55.56)		
Total	132 (16.60)	665 (83.40)		

^a^Continuity Adjusted Chi-Square was applied because 25% of the cells have expected counts less than 5

In the multivariable logistic regression analysis, after adjusting for potential confounding factors, IPV was significantly associated with prenatal depression. Pregnant women who had experienced IPV were 2.50 times more likely to report prenatal depression (OR = 2.50, 95% CI: 1.60–3.90). In terms of the type of IPV, psychological abuse (OR = 2.04, 95% CI: 1.19–3.49), psychological and economic abuse (OR = 6.16, 95% CI: 1.48–25.58), and psychological and physical abuse (OR = 21.81, 95% CI: 5.23–91.04) were significantly associated with prenatal depression (Table 3).

**Table 3:** Association between domestic violence during pregnancy and prenatal depression

Variables	Model 1*		Model 2*	
	OR (95% CI)	*P* value	OR (95% CI)	*P* value
Overall IPV	2.50 (1.60, 3.90)	<.0001	–	–
Subtypes				
Psychological abuse	–	–	2.04 (1.19, 3.49)	0.0092
Psychological+ economic abuse	–	–	6.16 (1.48, 25.58)	0.0123
Psychological+ physical abuse	–	–	21.81 (5.23, 91.04)	<.0001

Model 1: Adjusted for maternal age, maternal education, maternal occupation, husbands’ education, husbands’ occupation, husbands’ drinking and smoking characteristics, pregnancy related complications and abortion history, overall domestic violence as an independent factor;

Model 2: Adjusted for maternal age, maternal education, maternal occupation, husbands’ education, husbands’ occupation, husbands’ drinking and smoking characteristics, pregnancy related complications and abortion history, subtypes of domestic violence as independent factors

After adjusting for confounding factors, both IPV and prenatal depression had a significant association with adverse birth outcomes; the adjusted ORs for these variables were 1.72 (OR = 1.72, 95% CI: 1.11–2.68) and 1.67 (OR = 1.67, 95% CI: 1.08–2.56), compared with women reporting no IPV and no prenatal depression, respectively (Table 4). Regarding IPV subtype, only psychological abuse had a significant association with adverse birth outcomes (OR = 2.13, 95% CI: 1.08–2.58).

**Table 4:** Association of domestic violence during pregnancy+ prenatal depression and adverse birth outcomes

Variables	Model 1*		Model 2*	
	OR (95% CI)	*P*	OR (95% CI)	*P*
Prenatal depression	1.72 (1.11, 2.68)	0.0161	1.76 (1.13, 2.73)	0.0124
Domestic violence	1.67 (1.08, 2.56)	0.0202	–	–
Only psychological abuse	–	–	2.13 (1.08, 2.58)	0.0026

Model 1: Adjusted for maternal age, maternal education, maternal occupation, husbands’ education, husbands’ occupation, husbands’ drinking and smoking characteristics, pregnancy related complications and abortion history, prenatal depression and overall domestic violence as independent factors;

Model 2: Adjusted for maternal age, maternal education, maternal occupation, husbands’ education, husbands’ occupation, husbands’ drinking and smoking characteristics, pregnancy related complications and abortion history, prenatal depression and subtypes of domestic violence as independent factors

## Discussion

In this study, the prevalence of IPV among pregnant women in Wuhan, China, was 18.32%. We observed a significant and positive association between IPV and prenatal depression among pregnant women. Moreover, both maternal IPV and prenatal depression were associated with adverse birth outcomes. The prevalence rate was similar to a previous study’s finding of an 18.8% prevalence of IPV among pregnant women in Hong Kong [15]. The prevalence rate was higher than a previous finding of 11.3% for overall prevalence of IPV among women during pregnancy in Changsha city [16]. Psychological abuse (14.6%) was the most common form of abuse, accounting for 64.38% of abuse experienced by the women in this study. This was followed by physical abuse (2.1%). This finding was consistent with a previous study in China, which found that psychological violence was the most common form of violence among pregnant Chinese women (59/96 = 61.5%) [16]. A previous study in Iran also found that psychological violence was the most common form of violence among pregnant women (51.3%) [34]. A study in Thailand reported that 54% of women had been exposed to emotional violence, 27% to physical violence, and 19% to sexual violence [35]. Further, a meta-analysis of 92 studies from 23 countries focusing on IPV among pregnant women reported the prevalence of domestic violence as 13.3% in developed countries, compared to 27.7% for developing countries (*P* = 0.14) [36].

We found that women who had experienced IPV during pregnancy had a higher prevalence of depressive symptoms than did those who had not experienced IPV (29.45% vs. 13.67%, *P* < 0.0001). After adjustment for women’s and their husbands’ demographic characteristics, smoking, and alcohol use, overall IPV was associated with an increased risk of prenatal depression (OR = 2.50, 95% CI: 1.60–3.90). Additionally, psychological abuse was significantly associated with prenatal depression (OR = 2.04, 95% CI: 1.19–3.49), and psychological abuse combined with economic abuse increased the odds of developing prenatal depression by six times (OR = 6.16, 95% CI: 1.48–25.58) compared to those no IPV and prenatal depression. Our findings confirmed that IPV could lead to maternal depression and to a variety of adverse pregnancy outcomes.

Previous work has found strong associations between IPV and adverse outcomes, including LBW and PTB among women [37, 38]. In this study, we found that IPV during pregnancy was significantly associated with an increased odds of adverse birth outcomes (OR = 2.50, 95% CI: 1.60–3.90). This finding was consistent with previous studies. In Iran, a significant association was found between IPV and preterm labor (OR = 1.54, 95% CI: 1.16–2.03) [13]. In Vietnam, exposure to IPV was found to be associated with an increased risk of PTB (OR = 13.3, 95% CI: 2.5–69.9) and an increased risk of LBW (OR = 9.4, 95% CI: 2.0–44.3) among pregnant women [20]. Similar results were found in the United States, where IPV exposure was shown to increase the odds of the prematurity (OR = 1.45, 95% CI: 1.29–1.62), LBW (OR = 1.57, 95% CI: 1.25–1.97), and respiratory problems (OR = 1.17, 95% CI: 1.04–1.32) [4]. A recent study in India using adjusted regression models also revealed a significant association between IPV and both miscarriage (OR = 1.35, 95% CI: 1.11–1.65) and stillbirth (OR = 1.36, 95% CI: 1.02–1.82) [39].

We also found that women exposed to different types of IPV had different effects on prenatal depression and adverse birth outcome. Previous studies found that exposure to psychological and sexual violence may influence the pregnancy through alterations in women’s psychological wellbeing and lifestyle habits. Poor psychological wellbeing, in turn, may lead to hypertension or preeclampsia, which may be associated with insufficient weight gain and PTB [40, 41]. We also found significant associations between psychological abuse and prenatal depression (OR = 2.04, 95% CI: 1.19–3.49) and between psychological abuse and adverse birth outcomes (OR = 2.13, 95% CI: 1.08–2.58). In addition, women experiencing psychological abuse combined with subtype of physical IPV or economic IPV had higher odds ratios of adverse birth outcomes, compared with women who were not exposed to abuse. Taken together, our results show that IPV exposure, especially to psychological abuse, was significantly associated with prenatal depression and with adverse birth outcomes. These findings were consistent with a previously conducted prospective cohort study of IPV during pregnancy and adverse pregnancy outcomes in Vietnam [20].

The present study had several limitations. First, because the data on IPV and depression during pregnancy were collected in a cross-sectional study, we cannot establish the temporal relation between these two conditions. Second, we did not consider experiences of IPV or depressive symptoms prior to pregnancy that might be a potential factor of prenatal depression. The separate and combined associations of these conditions before and during pregnancy with birth outcomes warrant further investigation. Third, we should be careful to generalize the data to the completely pregnant population because the finding was obtained from a city of central China.

## Conclusions

This study has shown that IPV and prenatal depression were common among pregnant women in Wuhan, China. IPV was significantly associated with prenatal depression, and both IPV and prenatal depressive symptoms were associated with risk increasing of adverse birth outcomes, which are deleterious to maternal and newborn outcomes. The findings suggested that screening for IPV and prenatal depressive symptoms during prenatal care is necessary and might be helpful in reducing adverse outcomes for both mothers and newborns.

## Abbreviations

AAS: The Abuse Assessment Screen
ABO: Adverse birth outcomes
aOR: Adjusted odds ratio
CES-D: The Center for Epidemiologic Studies-Depression scale
CI: Confidence interval
IPV: Intimate partner violence
LBW: Low birth weight
PTB: Preterm birth
WHO: World Health Organization
